# Crisp and quiet: A novel programmable transcriptional repressor in plants

**DOI:** 10.1093/plphys/kiae211

**Published:** 2024-04-09

**Authors:** Sara Selma

**Affiliations:** Assistant Features Editor, Plant Physiology, American Society of Plant Biologists; VIB Center for Plant Systems Biology, 9052 Ghent, Belgium

Just as a well-designed machine performs various tasks through its interconnected components, plants can act as sophisticated systems in terms of survival, adaptability, and production. In that sense, it is important to have molecular tools that allow us to specifically “turn on or off” genetic expression, optimizing the responses of plants to different stimuli. The arrival of CRISPR (Clustered Regularly Interspaced Short Palindromic Repeats) systems allows us to expand and create new functionalities for the molecular toolbox available for plants. The CRISPR/Cas9 is comprised of 2 essential components: a Cas9 protein (CRISPR-associated protein9) and a small guide RNA (gRNA) that drives the Cas9 protein to the target sequence. This CRISPR/Cas9 complex causes a double-stranded DNA break in the target sequence and thus specific modifications in the genome (Cong et al. 2013). Although this tool was born as a powerful genomic editing system, the versatility to manipulate the structure of its components has allowed it to expand its applications. For example, by using the nuclease-dead Cas9 (dCas9), which maintains its RNA-directed DNA binding, and attaching transcriptional regulators in its structure it is possible to regulate the gene expression of the target genes (Gilbert et al. 2014). These programmable transcriptional regulators have been successfully developed for plants, obtaining a diverse battery of strong and specific CRISPR-based transcriptional activators (CRISPR activation or CRISPRa) (Pan et al. 2021). However, the obtention of efficient transcriptional repressors based on CRISPR (CRISPRi) has not been fully accomplished in plants. Although some examples of CRISPRi in plants have been reported, such as dCas9-BRD, dCas9-3xSRDX (SUPERMAN Repression Domain X), and dCas9-SRDX, the levels of repression achieved were not potent enough or had a strong target-dependent effect (Lowder et al. 2015; Piatek et al. 2015; Vazquez-Vilar et al. 2016).

In this issue of *Plant Physiology*, Wang et al. present the dCas9-N, a novel programmable transcriptional repressor based on CRISPR. This CRISPRi strategy showed efficient transcriptional repression of the target genes in Arabidopsis (*Arabidopsis thaliana*) and, for the first time, cucumber (*Cucumis sativus*).

The authors’ initial strategy was to fuse the TEN protein, characterized in cucumber as a noncanonical histone acetyltransferase (HAT), to dCas9 to increase the target gene expression in cucumber (Yang et al. 2020). Two gRNAs (T1 and T4) were designed to target the cucumber gene *CsACO1*, whose transcriptional perturbation affects tendril development, causing an easily identifiable phenotype (Fig.). In parallel, to test the dCas9-TEN, the CRISPRa strategy based on the fusion of the mammalian acetyltransferase P300 to the dCas9 was evaluated together with a dCas9-HA control, which does not contain any transcriptional regulation domain attached. Surprisingly, instead of observing transcriptional activation, the results show the transcriptional activity of the *CsACO1* gene had been repressed by 92% when it was targeted with the dCas9-TEN and the T4 gRNA.

**Figure.**
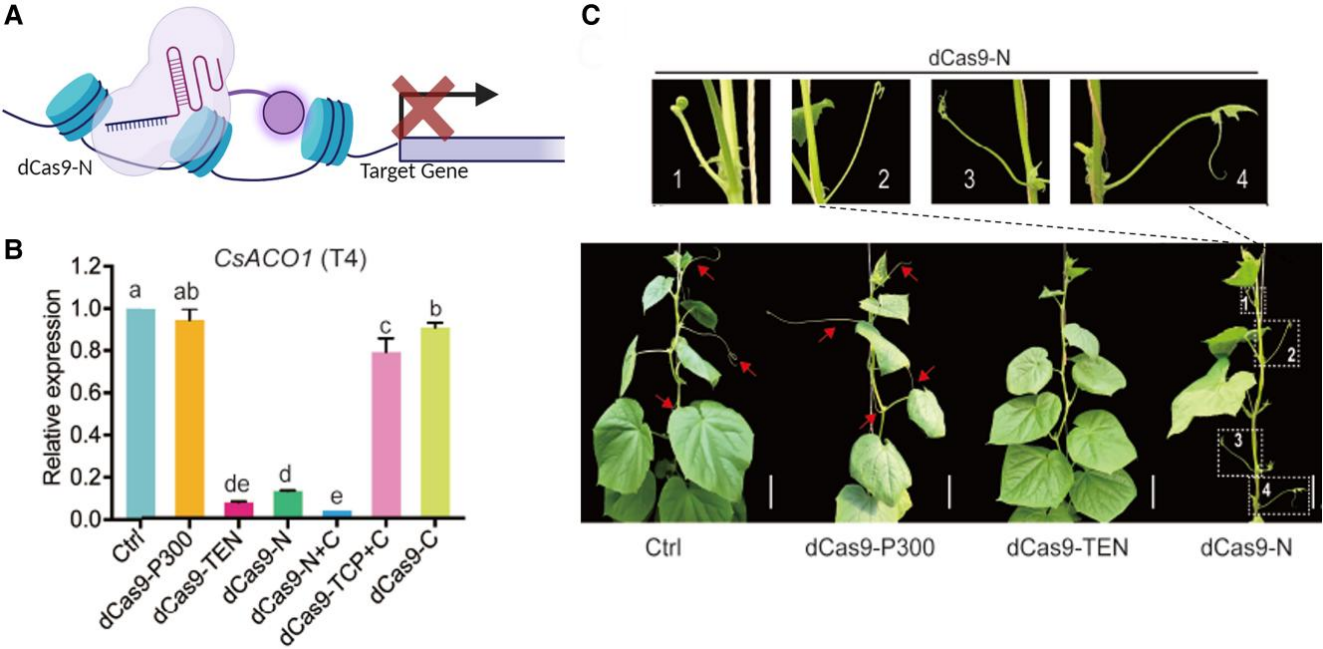
The dCas9-N fusion protein as a novel programmable transcriptional repressor. **A)** A schematic illustration of action mode of dCas9-N, binding the DNA assembled in the nucleosome and repressing the target gene expression. The illustration was generated with Biorender.com**B)** Relative *CsACO1* expression indicates that the N domain is the main transcriptional inhibitory domain of TEN. **C)** Representative photographs showing the phenotype of different transgenic plants harboring the indicated vector transformed in cucumber. All dCas9 variants target the T4 site of *CsACO1*. The tendril-less or modified tendril phenotype can be observed for the targeted plants with the Cas9 variants fused to the N domain. Scale bars, 10 cm; red arrows point to normal tendrils. B and C were extracted from the Wang et al. 2024.

Given the unexpected but remarkable result obtained with the dCas9-TEN strategy, the authors decided to investigate the transcriptional effects of the TEN protein and optimize it as a novel transcriptional repressor. The authors fused different truncated versions of the TEN protein to dCas9 to evaluate the roles of each domain (N, TCP, and C) in transcriptional repression. The results of the evaluation of the TEN domains, individually and in combination, reveal that TEN without the N domain showed weak repression, indicating that the N domain is the effector domain that modulates the transcription. The fusion of dCas9 with the N domain (dCas9-N) was used for subsequent experiments (Fig.). Additional experiments employing more gRNA to target different positions of the gene demonstrate that the design of the gRNA is a determinant step for achieving strong transcriptional repression. The positions close to transcription initiation or elongation are significantly hindered by the binding of dCas9-N, but those positions far from the transcriptional start site are less effective. To test the versatility of the dCas9-N, an evaluation of the transcriptional repression capacity was carried out over various genes across different basal expression levels. The results showed that dCas9-N efficiently repressed gene expression more than 50% across all target sites, indicating its robustness and versatility.

The transcriptional repression capacity was also tested in Arabidopsis both in transient expression and transgenic lines. Both dCas9-N and N-dCas9, the latter containing the protein N fused in the N terminal, had repression efficiencies similar to the reported dCas9-3SDRX when the *AtFL2* gene was targeted. The transgenic plants generated targeting the *AtFL2* gene with an N-dCas9 showed a compromised reactive oxygen species production in response to flagellin (flg22), validating the repression results obtained in Arabidopsis.

The next question to address regarding the novel CRISPRi tool is its specificity. To solve this requirement, the authors evaluated genome-wide transcriptional repression specificity using qRT-PCR and RNA-seq, confirming effective downregulation of the 6 target genes and showing that gene expression at the whole genome level is not broadly influenced by N-dCas9/dCas9-N in both cucumber and Arabidopsis. Also, the potential off-target sites of gRNAs were identified and examined, showing minimal impact on off-target genes.

Finally, to understand the effect of N protein in the chromatin and thus its repression capacity, the authors conducted an experiment with an in vitro production of histones followed by a nucleosome assembly with a free DNA probe. An electrophoretic mobility shift assay (EMSA) for detecting protein-nucleic acid interactions was performed including the purified N protein. The results of the EMSA demonstrate the binding of the N protein to DNA in nucleosomes, suggesting that the N protein's mode of action is to block the transcription process.

In summary, Wang et al. present a novel CRISPR-based programmable transcriptional repressor for plants. For the first time, a CRISPRi strategy was developed in cucumber, obtaining remarkably targeted gene repression. However, the repression capability of the dCas9-N was also evaluated in other species, demonstrating its orthogonality. Further optimizations of the dCas9-N can be also performed in the future to increase its repression capacity—for example, adapting the N protein in CRISPR strategies to recruit more regulation domains, such as the SunTag the scRNA strategy (Pan et al. 2021), or employing alternative Cas proteins, such as dCas12a, to have more versatility in the gRNA design.
